# Anti-Inflammatory Effects of Geniposidic Acid on *Porphyromonas gingivalis*-Induced Periodontitis in Mice

**DOI:** 10.3390/biomedicines10123096

**Published:** 2022-12-01

**Authors:** Tetsuya Tamura, Ruoqi Zhai, Tasuku Takemura, Kazuhisa Ouhara, Yuri Taniguchi, Yuta Hamamoto, Ryousuke Fujimori, Mikihito Kajiya, Shinji Matsuda, Syuichi Munenaga, Tsuyoshi Fujita, Noriyoshi Mizuno

**Affiliations:** 1 Department of Periodontal Medicine, Graduate School of Biomedical and Health Sciences, Hiroshima University, 1-2-3 Kasumi, Minami-ku, Hiroshima 734-8553, Japan; 2 Department of Innovation and Precision Dentistry, Graduate School of Biomedical and Health Sciences, Hiroshima University, 1-2-3 Kasumi, Minami-ku, Hiroshima 734-8553, Japan; 3 Department of General Dentistry, Hiroshima University Hospital, 1-2-3 Kasumi, Minami-ku, Hiroshima 734-8553, Japan

**Keywords:** bone resorption, geniposidic acid, interleukin 6, mouse, *Porphyromonas gingivalis*

## Abstract

Periodontal disease is predominantly caused by the pathogenic bacterium *Porphyromonas gingivalis* that produces inflammation-inducing factors in the host. *Eucommia ulmoides* is a plant native to China that has been reported to reduce blood pressure, promote weight loss, and exhibit anti-inflammatory effects. Geniposidic acid (GPA) is the major component of *E. ulmoides*. Herein, we investigated the effects of GPA on *P. gingivalis*-induced periodontitis by measuring the inflammatory responses in human gingival epithelial cells (HGECs) after *P. gingivalis* stimulation and GPA addition in a *P. gingivalis*-induced periodontitis mouse model. We found that GPA addition suppressed interleukin (IL)-6 mRNA induction (33.8% suppression), IL-6 production (69.2% suppression), toll-like receptor (TLR) 2 induction, and mitogen-activated protein kinase (MAPK) phosphorylation in HGECs stimulated by *P. gingivalis.* Inoculation of mice with GPA inhibited *P. gingivalis*-induced alveolar bone resorption (25.6% suppression) by suppressing IL-6 and TLR2 production in the serum and gingiva. GPA suppressed osteoclast differentiation of bone marrow cells induced by M-CSF and sRANKL in mice (56.7% suppression). GPA also suppressed the mRNA expression of OSCAR, NFATc1, c-Fos, cathepsin K, and DC-STAMP. In summary, GPA exerts an anti-inflammatory effect on periodontal tissue and may be effective in preventing periodontal disease.

## 1. Introduction

Periodontal disease is an inflammatory condition caused by the immune system in response to interactions with periodontopathogenic bacteria [1]. *Porphyromonas gingivalis*, a red complex bacterium, is an example of one causative bacterium and produces various pathogenic factors, such as gingipain, fimbriae, lipopolysaccharide (LPS), and volatile sulfur compounds [2]. Gingival epithelial cells act as the first line of defense toward eliminating bacterial challenges by producing inflammatory cytokines [3]. However, severe immune responses disrupt the epithelial defense line, allowing *P. gingivalis* to invade the periodontal tissue [4]. Further inflammation follows, and osteoclast activation by recombinant receptor activator of nuclear factor kappa-B ligand (RANKL) and bone resorption are observed. Finally, teeth fall out, causing masticatory disorders [5]. Recently, *P. gingivalis* was shown to be hematogenously transmitted to other organs, causing some systemic diseases, including non-alcoholic steatohepatitis and rheumatoid arthritis, and is associated with premature birth, low-weight birth, Alzheimer’s disease, and Buerger disease [6,7,8,9,10]. Patients with these systemic diseases often have difficulty with self-care. Therefore, a simple method for preventing periodontitis is needed, particularly considering that the prevalence of periodontitis increases with an aging society bearing older teeth.

Irsogladine maleate (IM) and glycyrrhizic acid (GLA), natural organic compounds abundant in licorice, were reported to suppress periodontitis. IM suppressed the production of interleukin (IL)-8 induced by *Aggregatibacter actinomycetemcomitans*, a periodontopathogenic bacteria, and recovered *A. actinomycetemcomitans*-induced reduction of the intercellular connection mediated by conexin43 in human gingival epithelial cells (HGECs) [11]. IM also suppressed toll-like receptor (TLR) 2-mediated IL-8 production in HGECs [12]. Furthermore, GLA inhibited bacteria-induced IL-6, IL-1β, and tumor necrosis factor (TNF)-α expression in mouse gingival tissue by suppressing high-mobility group box 1 [13]. In addition, epigallocatechin gallate (EGCG), a component of green tea, exerted antibacterial and anti-inflammatory effects in *P. gingivalis*-induced inflammation by regulating TLR2/4 and inflammasome signaling [14]. Furthermore, natural compounds in tea can prevent periodontitis with few side effects and high safety [15].

Geniposidic acid (GPA) is a natural component of the iridoid glucoside extract from *Eucommia ulmoides* Oliv. Bark, which has been used as a natural medicine for thousands of years. Previous studies have shown that GPA has various pharmacological effects, including anti-inflammatory, anti-angiocardiopathic, and anti-neurodegenerative effects. In vitro, an extract from E. ulmoides Oliv. Bark acted as a neuroprotective agent to inhibit cytotoxicity by suppressing neuronal cell death in H_2_O_2_-induced SH-SY5Y neuroblastoma cells and LPS-induced inflammation and reactive oxygen species in BV-2 microglial cells [16,17]. Furthermore, GPA inhibited the progression of Alzheimer’s disease by preventing amyloid β-induced cytotoxicity [18]. Based on these results, GPA shows potential as a multitarget candidate for treating inflammatory diseases. Although GPA is absorbed through the oral cavity, its effect on the oral mucosa has not been reported. Therefore, measuring saliva and gingival crevicular fluid (GCF) biomarkers has been considered effective to examine the effects of GPA on various oral mucosal diseases [19].

Considering the results of previous studies and that tea is consumed orally, we hypothesized that GPA plays an anti-inflammatory role in the progression of periodontal disease. Therefore, we investigated the effect of GPA on *P. gingivalis* infection in frontline gingival epithelial cells against periodontopathic bacteria in a mouse periodontitis model. This study provides a foundation for clinical studies on the effectiveness of GPA as a natural food that can suppress periodontal disease.

## 2. Materials and Methods

### 2.1. GPA

GPA was purchased from Sigma-Aldrich (L9634, St. Louis, MO, USA). It was dissolved in phosphate-buffered saline (PBS) and used for in vitro and in vivo experiments. The concentration of GPA used in this study was confirmed to have no cytotoxicity, as previously described [17].

### 2.2. Cell Culture and Stimulation of HGECs by P. gingivalis

HGECs were cultured according to the manufacturer’s protocol (PCS-200-014, American Type Culture Collection (ATCC), Manassas, VA, USA). The cells were sub-cultured in Dermal Cell Basal Medium (PCS-200-030, ATCC, Manassas, VA, USA) supplemented with Keratinocyte Growth Kit (PCS-200-040, ATCC, Manassas, VA, USA) and 100 U penicillin/streptomycin (P4333-100ML, Sigma-Aldrich, St. Louis, MO, USA) at 37 °C in humidified air with 5% CO_2_. A confluent culture of HGECs was stimulated with *P. gingivalis* (10% formalin-fixed, strain 3327, ATCC, Manassas, VA, USA) (107 colony-forming units/mL) for 12 h to examine mRNA expression. As a negative control, HGECs were cultured without bacterial stimulation. *P. gingivalis* treatment was performed as previously described [20].

Bone marrow mononuclear cells (BMMCs) were collected from the femurs and tibias of C57BL/6J Jcl mice by density gradient centrifugation with Histopaque-1083 (Sigma-Aldrich, St. Louis, MO, USA) in complete alpha-modified Eagle’s minimum essential medium (Sigma-Aldrich), containing 10% fetal bovine serum (Invitrogen, Carlsbad, CA, USA), L-glutamine (Sigma-Aldrich, St. Louis, MO, USA), and antibiotics (penicillin, streptomycin, and gentamicin; Invitrogen, Carlsbad, CA, USA).

### 2.3. RNA Extraction and Quantitative PCR Analysis

After stimulation of HGECs or BMMCs, total RNA was isolated using an RNAiso Plus (TAKARA Bio, Tokyo, Japan) according to the manufacturer’s protocol. Briefly, 1 µg of total RNA was reverse-transcribed using ReverTraAce^®^ for RT-PCR (TRT-101, TOYOBO, Osaka, Japan). Then, cDNA (1 μL) of the reverse-transcribed samples was used for quantitative PCR under previously described amplification conditions [21] in a StepOnePlus device (Applied Biosystems, Waltham, MA, USA). Template cDNA (1 μL) was mixed with the Core Reagent Fast SYBR^®^ Master Mix system (4 μL, Applied Biosystems, Waltham, MA, USA), ultrapure water (4.5 μL), and primers (10 pmol each, 0.5 μL). The sequences of the primer pairs used for quantitation of IL-6; TLR2; OSCAR; nuclear factor of activated T cells, cytoplasmic 1 (NFATc1); c-Fos; cathepsin K (CatK); matrix metalloproteinase-9 (MMP-9); DC-STAMP; and β-actin mRNA expression are listed in supplemental Table S1. The primer sequences are based on previous reports [20,22,23].

### 2.4. Detection of Phosphorylated Proteins

Sandwich enzyme-linked immunosorbent assay (ELISA) kits (p38: PEL-P38-T180-T, JNK: PEL-JNK-T183-T, and ERK: PEL-Erk-T202-T, RayBiotech Life, Inc., Peachtree Corners, GA, USA) were used to measure the levels of human phosphorylated proteins in HGECs. Briefly, the samples were lysed with cell lysate buffer containing 1% proteinase inhibitor cocktail (Nacalai Tesque, Tokyo, Japan, 04080-11) and 0.1% phenylmethanesulfonylfluoride (Sigma-Aldrich, St. Louis, MO, USA). These samples were added to an immobilized antibody-coated plate and incubated for 2.5 h at 25 °C. After washing four times with PBS containing 0.05% Tween20 (PBST), rabbit anti-phosphorylated protein antibody was added to the wells to detect phosphorylated p38, JNK, or ERK. To detect total phosphorylated protein, anti-pan-p38 antibody, anti-pan-JNK antibody, anti-pan-ERK antibody, or anti-pan-NF-κB antibody was added to the wells and incubated for 1 h at 25 °C. After removing unbound antibodies by washing, horseradish peroxidase (HRP)-conjugated anti-rabbit immunoglobulin G (IgG) was pipetted into the wells. The wells were rewashed, tetramethylbenzidine substrate solution was added, and color development occurred in proportion to the amount of phosphorylated protein or total protein. A stop solution was added, and the intensity of the color was measured at 450 nm using an ELISA reader (OD405, Varioskan LUX, Thermo Fisher Scientific, Waltham, MA, USA). To analyze the amount of phosphorylated protein, the ratio of phosphorylated protein to the total non-phosphorylated protein was calculated for each sample and expressed as a ratio to the control (no stimulation).

### 2.5. Generation of P. gingivalis-Induced Experimental Periodontitis Mouse Model

All animal experimental procedures were approved by the Ethics Committee of Hiroshima University (approval No. A21-104). Briefly, in total, 24 C57BL/6J Jcl mice (8 weeks old; CLEA Japan, Inc., Tokyo, Japan) were maintained under specific pathogen-free conditions until inoculation with bacteria in a controlled climate (temperature 22–24 °C, humidity 40–60%) under a 12 h light/dark cycle. The mice were fed regular chow that had been subjected to gamma irradiation (Type MF; Oriental Yeast Co., Ltd., Tokyo, Japan), and all animal experiments were conducted in accordance with the ARRIVE guidelines (https://arriveguidelines.org/arrive-guidelines accessed on 12 December 2021). The mice were randomly divided into four groups (six mice per group, *n* = 6) for each experiment (control group: carboxymethyl cellulose (CMC) inoculation, GPA: GPA oral inoculation, Pg: *P. gingivalis* oral inoculation, Pg/GPA: GPA and Pg oral inoculation). *P. gingivalis* was inoculated with 10^8^ bacterial cells/50 μL in 2% CMC solution twice per week for six weeks. The same volume of CMC solution was used as the negative control. GPA diluted in CMC (50 mg/50 μL in 2% CMC) was also inoculated for 30 min before *P. gingivalis* or CMC inoculation.

### 2.6. Evaluation of Alveolar Bone Level in Mouse

The evaluation of alveolar bone level in mice was performed using Kawai’s method as described previously [24]. Briefly, the upper molar jaw was stained with methylene blue (Sigma-Aldrich, St. Louis, MO, USA) for 10 min and washed three times with PBS. We measured the length of the blue-stained root of all molar teeth, from the top of the alveolar bone to the enamel–cement junction. Differences between the treated and control mice (no treatment) were evaluated.

### 2.7. Histological Examination

The upper jaws of the mice were collected at the end of the experiment (6 weeks) and maintained in 4% paraformaldehyde for 48 h, kept in 10% ethylene diamine tetrameric acid for 14 days to decalcify, and embedded in paraffin. Tissue sections (7 μm-thick) were stained with hematoxylin and eosin (HE) [23]. The severity of inflammation was scored using a previously published criteria [25].

### 2.8. Detection of IL-6 Levels in HGECs Supernatant and Mouse Serum

To examine the effects of GPA, the supernatant of the HGECs’ culture medium was collected, and IL-6 content was measured using Human IL-6 ELISA MAX™ Deluxe (9430504, BioLegend, San Diego, CA, USA), as described previously [20]. Briefly, a solid-phase anti-IL-6 monoclonal antibody was diluted in coating buffer (1 μg/mL) and coated onto a 96-well ELISA plate (Sigma-Aldrich, St. Louis, MO, USA) to capture the target. After blocking each well with 1% bovine serum albumin in PBST, the standard or supernatant (diluted in assay buffer from 1 to 0 ng/mL) was applied to each well. After detection of antibody application (diluted in assay buffer to 1 μg/mL), HRP-conjugated anti-IgG (2000-fold dilution in assay buffer) was applied to the wells. Colorimetric reactions were developed with o-phenylenediamine (Sigma-Aldrich, St. Louis, MO, USA) in the presence of 0.02% H_2_O_2_. Color development was stopped by adding stop solution and measured using an ELISA reader. The actual target concentration was calibrated by referring to a standard curve prepared using serial dilutions. Each sample was examined in triplicate in a 96-well ELISA plate.

Serum and tissues from the gingiva were isolated from each mouse and homogenized using a cool mill (TK-CM20S, Tokken, Inc., Chiba, Japan) in RIPA Lysis and Extraction Buffer (89900, Thermo Fisher Scientific, Waltham, MA, USA, 100 mg tissue/100 μL) containing 1% proteinase inhibitor cocktail and 0.1% phenylmethanesulfonylfluoride. The serum samples were diluted two-fold with assay buffer. The levels of IL-6 in the supernatants of the tissue homogenates and sera were measured using ELISA (431304; BioLegend, San Diego, CA, USA) as previously described [26].

### 2.9. Western Blotting Analysis

Western blotting was performed to evaluate the production of TLR2 in HGECs. The cultured cells were lysed using 100 μL of 1× lysis buffer (Thermo Fisher Scientific, Waltham, MA, USA) containing 1% proteinase inhibitor cocktail and 0.1% phenylmethanesulfonylfluoride. The samples were electrophoresed on a 10% sodium dodecyl sulfate-polyacrylamide gel and then electrically transferred onto nitrocellulose membranes (Bio-Rad Laboratories, Hercules, CA, USA). The membranes were blocked with 1% nonfat dried milk at 25 °C for 1 h and then incubated with anti-TLR2 monoclonal IgG (sc-166900-HRP, 10 μg/mL; Santa Cruz Biotechnology, Dallas, TX, USA) or anti-TLR2 polyclonal IgG (ab213676, 10 μg/mL; Abcam, Cambridge, UK) (ab213676) in PBS-Tween 20 at 4 °C for 12 h. For detection of anti-TLR2 polyclonal IgG, the membrane was incubated with HRP conjugated with goat anti-rabbit IgG (HAF008, R&D Systems, Inc., Minneapolis, MN, USA) in PBST at room temperature for 1 h. An anti-actin monoclonal antibody (sc-47778-HRP, 10 μg/mL; Santa Cruz Biotechnology) was used as the internal control. Immunodetection was performed according to the instructions of the ECL Plus Western blotting kit (GE Healthcare Life Sciences, Little Chalfont, UK) [24].

### 2.10. Osteoclast Differentiation Assay

BMMCs collected from mouse bone marrow were seeded into 96-well plates at a density of 2 × 10^5^ cells/well in 15% fetal bovine serum-containing alpha-modified Eagle Minimum Essential Medium. BMMCs were cultured for 7 d with 100 ng/mL murine RANKL (577102, BioLegend, San Diego, CA, USA) and 20 ng/mL murine macrophage colony-stimulating factor (M-CSF, 576402, BioLegend, San Diego, CA, USA) to evaluate osteoclastogenesis. The medium was replaced with fresh medium containing soluble RANKL (sRANKL) and M-CSF and incubated for another 3 d. The cells were stained using a TRAP staining kit (TRAP/ALP Stain Kit, FUJIFILM Wako Chemicals, Tokyo, Japan). TRAP-positive (TRAP+) cells with three or more nuclei were considered as osteoclasts. TRAP+ multinuclear cells were counted, and the results were expressed as the number of positive cells per well in a 96-well plate. Murine sRANKL and M-CSF were dissolved in PBS for subsequent in vitro experiments. 

### 2.11. Statistical Analysis

All experiments were performed independently at least three times. Data are expressed as the mean ± standard deviation (SD). The Shapiro–Wilk test was conducted for each data set to evaluate the normality of the distribution. Statistical analyses between two groups were performed using two-tailed unpaired Student’s t-test. One-way analysis of variance with Tukey–Kramer post hoc tests were used for multiple comparisons. Statistical significance was set at *p* < 0.05.

## 3. Results

### 3.1. IL-6 and TLR2 Expression in HGECs

**Figure 1:**
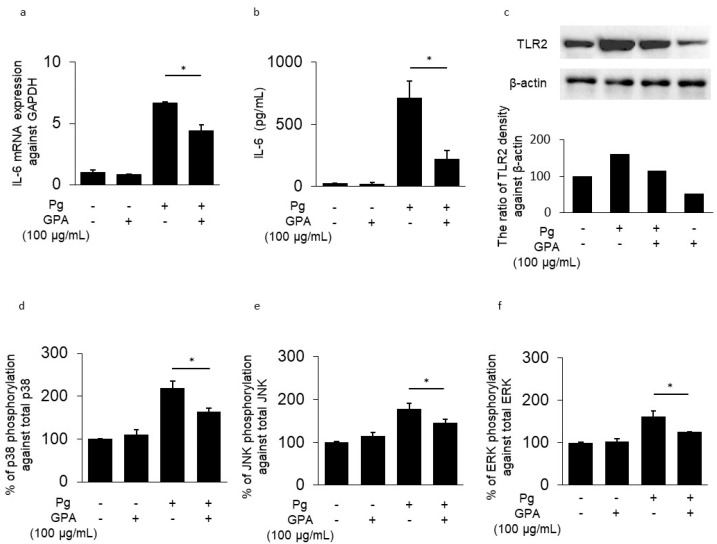
Inhibitory effects of geniposidic acid (GPA) on human gingival epithelial cells (HGECs) (**a**) mRNA expression of IL-6 in HGECs with or without *Porphyromonas gingivalis* stimulation (12 h) in the presence or absence of GPA (100 μg/mL). (**b**) Levels of IL-6 in the supernatant of HGECs with or without *P. gingivalis* stimulation (24 h) in the presence or absence of GPA (100 μg/mL). (**c**) Production of TLR2 in HGECs with or without *P. gingivalis* stimulation (12 h) in the presence or absence of GPA (100 μg/mL). (**d**–**f**) Levels of phosphorylated protein in HGECs with or without *P. gingivalis* stimulation (10 min) in the presence or absence of GPA (100 μg/mL, (**d**): p38, (**e**): JNK, f: ERK). Results are shown as the mean ± SD. * Significantly higher expression by Student’s *t*-test (* *p* < 0.01).

The mRNA expression of IL-6 was induced in HGECs by *P. gingivalis* stimulation (Figure 1a). This effect was suppressed by GPA addition (34% inhibition). The addition of only GPA to HGECs showed no effect on IL-6 mRNA levels compared to that in the control. *P. gingivalis* stimulation increased IL-6 protein production in the supernatant of HGECs. In contrast, GPA addition inhibited *P. gingivalis*-induced IL-6 production (69% inhibition) (Figure 1b). TLR2 production in HGECs was also upregulated by stimulation with *P. gingivalis*; this effect was suppressed by GPA treatment. The addition of only GPA in HGECs only slightly affected TLR2 suppression (Figure 1c). To identify the target molecules of GPA, phosphorylated proteins in HGECs were measured using ELISA. The levels of phosphorylated p38, JNK, and ERK proteins were increased following *P. gingivalis* stimulation for 10 min but were suppressed by the addition of GPA (p38: 25.0%, JNK: 18.6%, ERK: 22.8% inhibition) (Figure 1d–f). The addition of only GPA did not induce phosphorylation in HGECs. These results indicate that GPA suppresses TLR2 via the downregulation of mitogen-activated protein kinase (MAPK) phosphorylation. Furthermore, suppression of TLR2 may result in the downregulation of IL-6.

### 3.2. Inhibitory Effect of GPA in P. gingivalis-Induced Mouse Periodontitis Model

**Figure 2:**
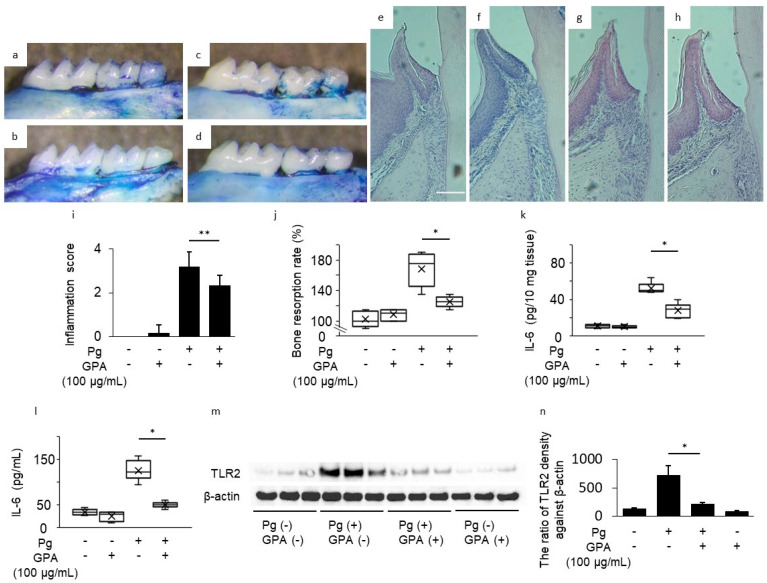
Inhibitory effects of geniposidic acid (GPA) on a *Porphyromonas gingivalis*-induced mouse periodontitis model. The mice were treated with *P. gingivalis* in the presence or absence of GPA and sacrificed, and the serum, gingival tissue, and upper molar bone were collected ((**a**) PBS inoculation; (**b**): GPA inoculation; (**c**): *P. gingivalis* inoculation; (**d**): GPA + *P. gingivalis* inoculation, *n* = 6 /group). Destruction of the mouse periodontal tissue was visualized in hematoxylin and eosin-stained sections (**e**): PBS inoculation, (**f**): GPA inoculation, (**g**): *P. gingivalis* inoculation, (**h**): GPA + *P. gingivalis* inoculation). Original magnification ×100; Scale bar = 100 µm. The inflammation score in sections from each group was evaluated (**i**). Data represents the mean ± SD of six mice per group. (**i**). Alveolar bone level of the upper jaw in each group (**j**). The amount of IL-6 in each group was determined using ELISA (**k**: gingival tissue, l: serum). In the boxplot, the horizontal black lines within each box denote the median values, and boxes are extended from the 25th and 75th percentile. Whiskers above and below the boxes indicate the 10th and 90th percentiles, respectively, and cross marks denote the averages (**j**–**l**). (**m**) Production of TLR2 in mouse gingival tissue of a representative sample (*n* = 3/group) with or without *P. gingivalis* inoculation in the presence or absence of GPA; the density of each band was measured by NIH imaging (**n**). Statistical analyses were performed using one-way ANOVA with Tukey–Kramer post hoc test (* *p* < 0.01, ** *p* < 0.05).

To assess the effect of GPA in vivo, we examined experimental periodontal disease in mice inoculated with *P. gingivalis* and control or GPA-treated gingival tissue, serum, and alveolar bone. Resorption of alveolar bone was increased in the Pg group compared to the control group; however, this increase was inhibited by oral administration of GPA in the Pg/GPA group (40% suppression, Figure 2a–d,j). Histological observations showed the same trend. The Pg group showed resorption in the alveolar bone and increased infiltration of inflammatory cells in the connective tissue compared to those in the control group (Figure 2e,g,i). However, GPA inhibited Pg-induced inflammation in the periodontal tissue (Pg/GPA group, Figure 2h,i). IL-6 levels in the gingival tissue and serum were elevated in the Pg group compared to those in the control group. This elevation was suppressed by oral administration of GPA to the Pg/GPA group (Figure 2k: gingival tissue, 39.4% suppression; Figure 2l: serum, 57.9% suppression). Single GPA addition had no effect compared to the control group. To clarify the target molecule of GPA in the gingival tissue, the protein level of TLR2 was determined using Western blotting. The increased TLR2 level in the Pg group was suppressed by oral administration of GPA in the Pg/GPA group (Figure 2m,n). These results show that orally administered GPA acts as an anti-inflammatory agent in periodontitis.

### 3.3. Inhibitory Effect of GPA in Osteoclast Differentiation

**Figure 3:**
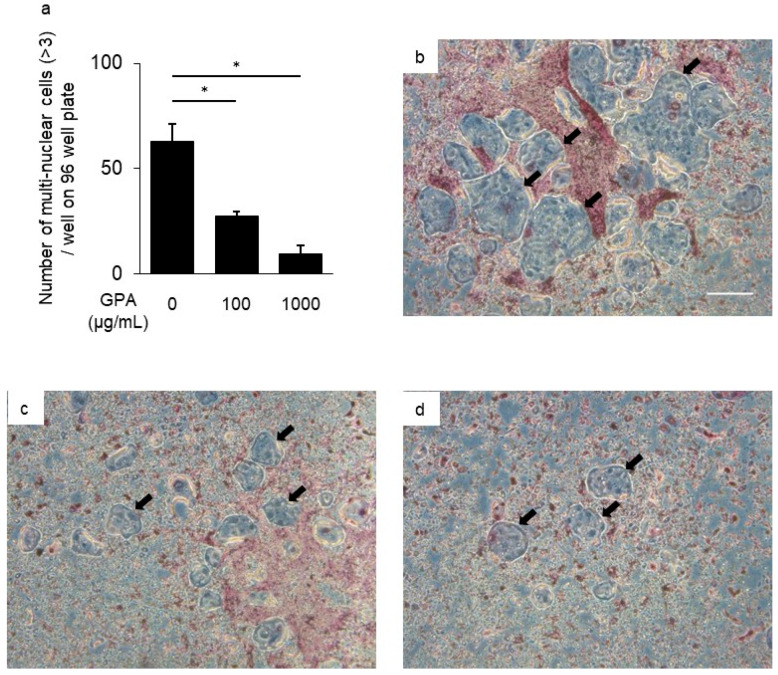
Inhibitory effect of geniposidic acid (GPA) on osteoclast differentiation of mouse BMMCs. BMMCs (2 × 10^5^ cells/well) were cultured on 96-well plates in the presence of sRANKL (100 ng/mL) and M-CSF (20 ng/mL) in the presence or absence of GPA ((**b**): 0 μg/mL, (**c**): 100 μg/mL, (**d**): 1000 μg/mL) for seven days. (**a**) Osteoclasts with multiple nuclei (>3) in 96-well plates were counted (black arrowheads). The data represent the results of three independent experiments. (**b**–**d**) Representative TRAP-stained images of multinuclear osteoclasts in 96-well plates on day 7. Original magnification ×100; Scale bar = 100 µm. Results are shown as the mean ± SD. * Significantly larger number of osteoclasts by Student’s *t*-test (* *p* < 0.01).

**Figure 4:**
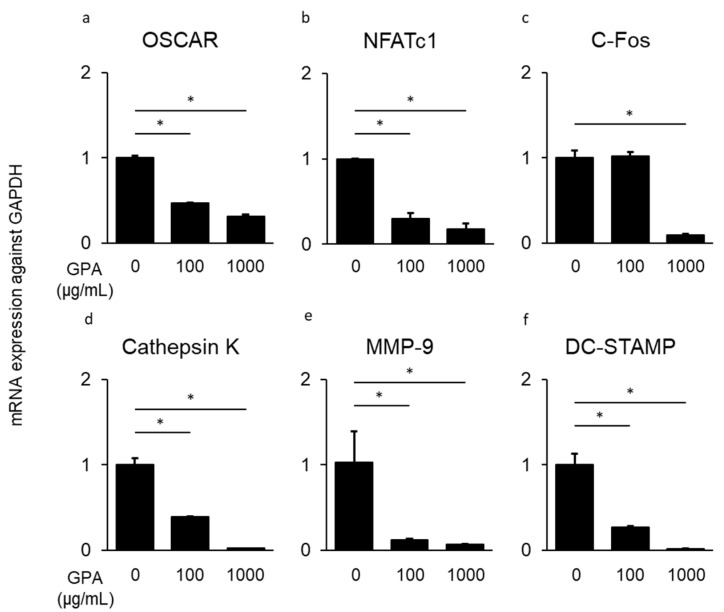
Inhibitory effect of geniposidic acid (GPA) on mRNA induction of osteoclast differentiation-related genes. To determine the effect of GPA in osteoclast differentiation, BMMCs were cultured with sRANKL (100 ng/mL) and M-CSF (20 ng/mL) for 48 h in the presence or absence of GPA (100 or 1000 μg/mL). mRNA expression was determined using quantitative PCR ((**a**): OSCAR, (**b**): NFATc1, (**c**): c-Fos, (**d**): cathepsin K, (**e**): MMP-9, and (**f**): DC-STAMP). * Significantly higher mRNA expression of genes by Student’s *t*-test (* *p* < 0.01).

**Figure 5:**
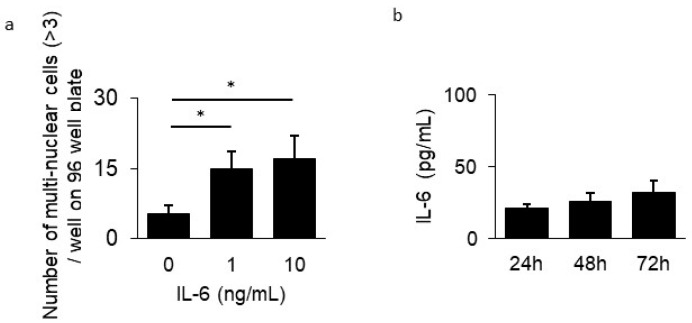
Effect of IL-6 on osteoclast differentiation. (**a**) Osteoclast differentiation assay was performed in the presence of sRANKL (100 ng/mL), M-CSF (20 ng/mL), and IL-6 (0, 1, 10 ng/mL) for 7 days. Osteoclasts with multiple nuclei (>3) in 96-well plates were counted after TRAP-staining. (**b**) IL-6 production in the supernatant of BMMCs in the presence of sRANKL (100 ng/mL) and M-CSF (20 ng/mL) after 24, 48, and 72 h was measured using ELISA. The data represent the results of three independent experiments. * Significantly larger number of osteoclasts by Student’s *t*-test (* *p* < 0.01).

To clarify the mechanism responsible for the suppressive ability of GPA on bone resorption in a mouse periodontal disease model, osteoclast differentiation from BMMCs was assessed following treatment with GPA. The number of multinuclear giant cells in BMMCs derived from mice treated with GPA was much lower than that from untreated mice (58.9% suppression, Figure 3a–d). The mRNA expression of genes related to osteoclast differentiation (OSCAR, NFATc1, c-Fos, cathepsin K, MMP-9, and DC-STAMP) in BMMCs treated with GPA, sRANKL, and M-CSF after 48 h was determined using quantitative PCR. The mRNA expression of these genes induced by sRANKL and M-CSF was suppressed following treatment with GPA in a concentration-dependent manner (OSCAR: 68.1%, NFATc1: 81.9%, c-Fos: 92.3%, cathepsin K: 97.8%, MMP-9: 92.7%, and DC-STAMP: 98.3% suppression, Figure 4a–f). In this present study, IL-6 production was suppressed by GPA. IL-6 has also been shown to affect osteoclast differentiation through a pathway that is not mediated by sRANKL and M-CSF [27]. This result is consistent with that of a previous study that showed that the presence of IL-6 with sRANKL and M-CSF induced osteoclast differentiation (Figure 5a). Therefore, to determine the production of IL-6 by BMMCs in the presence of sRANKL and M-CSF, IL-6 levels in cultured BMMCs were measured. IL-6 production was not increased in the supernatant of BMMCs cultured with sRANKL and M-CSF (Figure 5b). These findings indicate that in BMMCs, GPA does not affect IL-6 production but regulates osteoclast differentiation directly through correlated genes.

## 4. Discussion

We investigated the inhibitory effect of GPA on *P. gingivalis*-induced IL-6 production in HGECs and *P. gingivalis*-induced bone resorption in a mouse model. To the best of our knowledge, the results showed for the first time that GPA had an anti-inflammatory effect on gingival epithelial cells in vitro and had inhibitory effects on periodontitis-induced inflammation and bone resorption in a mouse periodontitis model. Furthermore, we found for the first time that GPA inhibits osteoclast differentiation in vitro. Periodontitis is an inflammatory disease caused by periodontopathogenic bacteria, including red complex bacteria such as *P. gingivalis*, *Tannerella fosythia*, and *Treponema denticola* [4]. HGECs act as the first line of defense against bacterial invasion [3]. TLRs play crucial roles in the innate immunity by recognizing pathogen-associated molecular patterns from various microbes [28]. TLRs expressed on the cell surface mainly recognize microbial membrane components such as lipids, lipoproteins, and proteins [29]. TLR1, TLR2, and TLR6 recognize a variety of pathogen-associated molecular patterns including lipoproteins, peptidoglycans, lipoteichoic acids, zymosan, mannan, and *Trypanosoma cruzi* trypomastigotes-mucin [30]. We found that treatment with GPA inhibited IL-6 production following TLR2 activation in HGECs. Whole *P. gingivalis* cells were used as a stimulator. *P. gingivalis* is strongly associated with various systemic diseases and bears surface components that can attach to host cells [2]. The *P. gingivalis* surface components fimbriae (Mfa1, FimA) and LPS are recognized by TLR2 and through downstream signaling [31,32]. Particularly, LPS derived from *P. gingivalis* (PgLPS) is a strong modulator of the immune response [33]. Because there have been reports of TLR2-mediated responses to PgLPS [34], further studies are needed to examine the effects of GPA using PgLPS. In addition, TLR2-mediated signaling following PgLPS stimulation has been reported. The NF-κB, MAPK, and AP-1 signaling pathways were shown to be involved in the production of inflammatory cytokines such as IL-6 and TNF-α via TLR2 recognition [30]. Among these signaling pathways, other studies showed that GPA affects MAPK phosphorylation, c-Jun activation, and IκB dephosphorylation [33]. The IL-6 suppression observed in this study may be mediated by the same pathway. Local application of GPA also suppressed IL-6 production in serum and gingival tissue and bone resorption in a *P. gingivalis*-induced mouse periodontitis model (Figure 2).

GPA treatment also suppressed IL-6 production in HGECs. These results indicate that locally applied GPA regulates inflammation in periodontal tissues. Furthermore, the inhibitory effect of GPA on periodontitis is thought to have a positive effect on the suppression of the exacerbation of systemic diseases that are affected by periodontal diseases such as diabetes, rheumatoid arthritis, and non-alcoholic steatohepatitis [6,7,8,9,10]. In addition, there are reports on the relationship between the severity of periodontitis and COVID-19 infection; therefore, it may affect the control of viral infection in the oral cavity, which is the gateway for entry of viruses [35]. However, it is unclear whether GPA had an anti-inflammatory effect locally in the periodontal tissue or whether it exerts this effect after being absorbed by the gastrointestinal tract and transferred to the periodontal tissue in a hematogenous manner. A previous study showed that application of GPA by daily oral gavage suppressed spatial learning and memory deficits and alleviated neuroinflammation by inhibiting high-mobility group box 1 and downregulating the TLR4/2 signaling pathway in APP/PS1 mice [36]. In our study, GPA was dissolved in highly viscous CMC, leading to its retention in the oral cavity. However, as GPA is water-soluble, it is easily absorbed by the mucosal surface; it may be absorbed both locally in the oral cavity and in the gastrointestinal tract, after which it exerts an anti-inflammatory effect in the periodontal tissue in a hematogenous manner. Whether the effects are local or occur through later absorption, GPA can be clinically ingested from the oral cavity and can be used to prevent periodontitis. Interestingly, GPA inhibits the differentiation of BMMCs into osteoclasts (Figure 3). As shown in Figure 5a, the addition of IL-6 induced osteoclast differentiation in vitro. However, IL-6 production was not increased in the osteoclast differentiation assay with sRANKL and M-CSF (Figure 5b). Previous studies have shown that activation of p38 and ERK phosphorylation in the MAPK signaling pathway, as well as downstream factors of NFATc1, c-Fos, and C-Jun are important [37]. As GPA suppresses activation of the MAPK, AP-1, and NF-κB signaling pathways in macrophages [38], alveolar bone resorption may occur in vivo through the indirect action of IL-6 suppression because of its anti-inflammatory effect and its direct effects on BMMCs.

This study had some limitations. First, the detailed regulatory mechanism of cytokine expression and osteoclast differentiation induced by GPA was not elucidated, requiring further analysis. Second, we did not confirm whether HGECs and BMMCs are targets of GPA for the downregulation of *P. gingivalis*-induced inflammation.

In conclusion, GPA downregulated IL-6 inflammatory responses in gingival tissue and improved alveolar bone loss by suppressing osteoclast differentiation. Further studies are needed to explore the clinical application of GPA and clarify the mechanism underlying its anti-inflammatory effects.

## Data Availability

All data generated or analyzed during this study are included in this published article.

## Supplementary Materials

The following supporting information can be downloaded at: https://www.mdpi.com/article/10.3390/biomedicines10123096/s1, Table S1: Primer sets used for real-time PCR analysis.
