# Supplementary material for: Anti-Inflammatory Effects of Geniposidic Acid on Porphyromonas gingivalis-Induced Periodontitis in Mice
Source: Biomedicines. 2022 Dec 1;10(12):3096. doi: 10.3390/biomedicines10123096 (PMC9775215; doi:10.3390/biomedicines10123096)
Supplement: Supplementary file 1 [file biomedicines-10-03096-s001.zip › biomedicines-2047621-supplementary.pdf]

Table S1. Primer sets used for real-time PCR analysis

Gene (protein)	Primer sequence (5′–3′)
Il-6 F (IL-6)	GGAGACTTGCCTGGTGAAAA
Il-6 R	GTCAGGGGTGGTTATTGCAT
Tlr2 F (TLR2)	GCCAAAGTCTTGATTGATTGG
Tlr2 R	TTGAAGTTCTCCAGCTCCTG
Oscar F (OSCAR)	TGGCGGTTTGCACTCTTCA
Oscar R	GATCCGTTACCAGCAGTTCCAGA
c-fos F (c-Fos)	AAGATGGCTGCAGCCAAGTG
c-foc R	TCCAGTTTTTCCTTCTCTTTCAGCA
Nfatc1 F (NFATc1)	TCATCCTGTCCAACACCAAA
Nfatc1 R	TTGCGGAAAGGTGGTATCTC
Mmp-9 F (MMP-9)	CTGGACAGCCAGACACTAAAG
Mmp-9 R	CTCGCGGCAAGTCTTCAGAG
Catk F (CatK)	GAAGAAGACTCACCAGAAGCAG
Catk R	TCCAGGTTATGGGCAGAGATT
Dc-stamp F (DC-STAMP)	CTTGCAACCTAAGGGCAAAG
Dc-stamp R	TCAACAGCTCTGTCGTGACC
β-actin F (β-actin)	GACGGGGTCACCCACACTGT
β-actin R	AGGAGCAATGATCTTGATCTTC
